# Elevated long-to-very-long-chain ceramide ratio correlates with disease severity in inflammatory bowel disease and primary sclerosing cholangitis

**DOI:** 10.1038/s41598-025-07308-8

**Published:** 2025-06-25

**Authors:** Tanja Elger, Muriel Huss, Gerhard Liebisch, Marcus Höring, Johanna Loibl, Arne Kandulski, Martina Müller, Hauke Christian Tews, Christa Buechler

**Affiliations:** 1 Department of Internal Medicine I, Gastroenterology, Hepatology, Endocrinology, Rheumatology, and Infectious Diseases, University Hospital Regensburg, 93053 Regensburg, Germany; 2 Institute of Clinical Chemistry and Laboratory Medicine, University Hospital Regensburg, 93053 Regensburg, Germany

**Keywords:** Calprotectin, Inflammatory bowel disease, Disease activity, Primary sclerosing cholangitis, Diagnostic markers, Gastrointestinal diseases, Biomarkers, Gastroenterology

## Abstract

There is strong evidence that ceramides play a significant role in the pathology of inflammatory bowel disease (IBD) and chronic liver injury. Long-chain (LC) and very long-chain (VLC) ceramides have opposing functions, yet the associations of circulating levels of ceramide species in patients with IBD and primary sclerosing cholangitis (PSC)—as inflammatory biliary-hepatic disease closely linked to IBD— with disease severity remain poorly studied. This study investigates whether serum levels of ceramide (Cer) and hexosylceramide, a glycated ceramide derivative, are associated with disease severity in these conditions. Serum levels of eight ceramide and five hexosylceramide species were measured in 16 healthy controls, 57 patients with IBD, 7 patients with PSC, and 13 patients with PSC-IBD. Lipid levels were determined using direct flow injection analysis with a triple quadrupole mass spectrometer. Patients with IBD exhibited higher levels of Cer 18:1;O2/16:0 and Cer 18:1;O2/18:0 compared to controls. Their LC/VLC ceramide ratio was elevated and positively correlated with C-reactive protein and fecal calprotectin. However, ceramide and hexosylceramide levels were not associated with stool consistency, disease localization, or extra-intestinal manifestations. Patients with PSC and PSC-IBD also had increased LC/VLC ceramide ratios, primarily due to a decline in VLC ceramide species. In PSC-IBD, this ratio correlated positively with cholestasis markers. Additionally, serum hexosylceramide 18:1;O2/16:0 and 24:1 levels were specifically elevated in PSC. This study demonstrates that an altered LC/VLC ceramide balance is associated with disease severity in IBD, PSC-IBD, and PSC, highlighting its potential as a biomarker for IBD, PSC-IBD, and PSC. As our PSC cohorts were small, a confirmatory study is required.

## Introduction

The etiology of inflammatory bowel disease (IBD) is complex and multifaceted, with numerous potential contributing factors, including genetics, stress, inflammation, gut microbiota dysbiosis, diet and toxins. Clinical symptoms of IBD include weight loss, diarrhea, abdominal pain, blood in the stool, and fatigue. Notably, IBD is an umbrella term that encompasses distinct phenotypes with Crohn’s disease (CD) and ulcerative colitis (UC) as the most important subtypes^1–4^.

Ceramides are precursors of more complex sphingolipids and have been the subject of considerable research^5–7^. Cellular ceramide levels are induced by lipopolysaccharide and tumor necrosis factor incubation, indicating a central function of these lipids in inflammation^3^. Ceramides are bioactive lipids that play a role in essential biological processes, including apoptosis, proliferation, insulin response and cell senescence^3,8,9^.

The biological effects of ceramides are defined by the acyl-chain length of their fatty acids. Long-chain ceramides (C16-C20) induce insulin resistance and cell death, whereas very long-chain ceramides (C22-C26) have the opposite effect^7,10,11^. Accordingly, long-chain ceramides are positively related to the risk of metabolic syndrome and cardiovascular diseases^12^. The Framingham Heart Study and the Study of Health in Pomerania revealed that the C24-ceramide-to-C16-ceramide ratio was inversely correlated with the risk of coronary heart disease, heart failure and overall mortality^13^.

The synthesis of the different ceramide species is catalyzed by ceramide synthases, of which six distinct isoforms have been characterised. Notably, mice with global knockdown of ceramide synthase 2 (synthesis of C22-24 ceramides), ceramide synthase 4 (synthesis of C18-20 ceramides), ceramide synthase 5 (synthesis of C14-16 ceramides), and ceramide synthase 6 (synthesis of C14-16 ceramides) all experienced more severe colitis^3,12,14–16^. Compared to controls, ceramide synthase 2 mRNA expression in the ascending colon of patients with UC was increased^17^, and this upregulation may be regarded as protective^3^. However, in IL-10-deficient mice with severe IBD, the knockdown of ceramide synthase 2 improved inflammation^18^, indicating proinflammatory activities of VLC ceramides in this model.

Neutral ceramidase is one of five different enzymes involved in the breakdown of ceramides^19^. Neutral ceramidase is abundant in the small intestine and colon^19^ and is highly expressed in the tissues of patients with UC and mice with dextran sulfate sodium (DSS)-induced colitis^20^. Global knockout of this enzyme unexpectedly increased sphingosine-1-phosphate levels and inflammation after DSS treatment, indicating a protective effect of neutral ceramidase in IBD^20^. Etrasimod, a drug that selectively activates the sphingosine-1-phosphate receptor subtypes 1, 4, and 5, is effective in patients with UC^21^, further suggesting a central and complex function of sphingolipids in IBD. The expression of acid ceramidase is elevated in the inflammatory cell infiltrates of patients and mice with colitis. Loss of this enzyme in myeloid cells results in reduced recruitment of neutrophils and lower levels of cytokines and chemokines, protecting mice from colitis^22^.

The multifaceted roles of sphingolipids are further compounded by the specific functions of hexosylceramides, which are produced by the modification of ceramides with glucose. Lipopolysaccharide induces glucosylceramide accumulation in murine dendritic cells and the kidney, spleen and liver of Syrian hamsters^23^. The levels of these lipids were generally increased in the serum of patients with inflammatory conditions^24^. It has also been shown that serum levels of hexosylceramides are negatively correlated with C-reactive protein and interleukin-6 in a cohort of middle-aged and older adults^25^, but the relationship between this lipid class and inflammation needs further study^23^.

Primary sclerosing cholangitis (PSC) is a rare, progressive liver disease characterized by bile duct fibrosis, leading to multifocal strictures and secondary liver cirrhosis. No causal therapy has been approved, and some patients ultimately require liver transplantation^26–28^. PSC is frequently associated with inflammatory bowel disease (PSC-IBD), yet the underlying pathology of PSC-IBD remains poorly understood^26–28^. Notably, in a mouse model, PSC was shown to attenuate the severity of IBD, and in patients, it has been linked to a milder disease course^29^.

In patients with PSC without underlying UC, ceramide synthase 1 mRNA expression was upregulated in the ascending colon compared to healthy controls, UC patients, and PSC-UC patients. In contrast, ceramide synthase 1 expression in the sigmoid colon was highest in PSC-UC^17^. Furthermore, in women with severe intrahepatic cholestasis of pregnancy, plasma levels of Cer 18:1/22:1, 23:0, and 24:0 were reduced, while Cer 18:1/24:1 was elevated^30^.

### Hypothesis and study objective

To our knowledge, it remains unknown whether similar alterations in serum ceramide levels occur in patients with PSC and PSC-IBD. Additionally, the associations of different ceramide and hexosylceramide species with inflammation among IBD patients have not been fully elucidated. This study aims to analyze serum levels of eight ceramide and five hexosylceramide species in healthy controls and patients with IBD, PSC, and PSC-IBD to assess their associations with intestinal inflammation and liver disease severity.

## Materials and methods

### Patients and controls

Patients with a confirmed diagnosis of inflammatory bowel disease (IBD) who were admitted to the Department of Internal Medicine I at the University Hospital Regensburg between June 12, 2021, and February 23, 2023, were invited to participate in this study. IBD was diagnosed based on endoscopic, histological, and clinical criteria^31,32^. Exclusion criteria included coagulopathy, pregnancy, and the inability to provide informed consent. Control subjects comprised spouses of patients, hospital employees, and students. All participants provided written informed consent before enrollment. The study was approved by the Ethics Committee of the University Hospital Regensburg (Protocol No. 19–1309-101, approval date: 20.02.2019) and conducted in accordance with the latest guidelines for good clinical practice and the updated Declaration of Helsinki.

### Lipid extraction and analysis of ceramide species

Lipids were isolated from serum samples (10 µL) following an established protocol^33^. Non-naturally occurring lipid species were used as internal standards and added before lipid extraction (Cer 18:1;O2/14:0, Cer 18:1;O2[D7]/18:0, HexCer 18:1;O2/12:0, HexCer 18:1;O2[D5]/18:0; Avanti Polar Lipids, AL, USA). For extraction, 2 mL of chloroform was used. Subsequently, 1 mL of the chloroform phase was separated, vacuum-dried, and dissolved in a methanol (Merck, Darmstadt, Germany)/chloroform (Roth, Karlsruhe, Germany) mixture (3:1, v/v) containing 7.5 mM ammonium acetate^34^. Ceramide and hexosylceramide species were analyzed using direct flow injection analysis (FIA) with a triple quadrupole mass spectrometer (FIA-MS/MS; QQQ triple quadrupole) in positive ion mode, detecting a fragment ion at m/z 264. Quantification was performed using calibration curves, as previously described^35^*.*

### Statistical analysis

Data are presented as boxplots and bar charts (mean ± standard deviation). Statistical analyses were performed using the Kruskal–Wallis test and Spearman’s correlation (IBM SPSS Statistics 26.0; IBM Corp., Armonk, NY, USA, released 2019).

To adjust for multiple comparisons, all *P*-values were multiplied by 13, corresponding to the number of lipid species analyzed in this study. A *P*-value of < 0.05 was considered statistically significant.

## Results

This study included 16 controls and 57 patients with inflammatory bowel disease (IBD), comprising 17 patients with ulcerative colitis (UC) and 40 patients with Crohn’s disease (CD). Additionally, seven patients had primary sclerosing cholangitis (PSC), and 13 had PSC-IBD. The control group was age-matched with the PSC group but was older than the IBD (*P* = 0.003) and PSC-IBD (*P* = 0.029) groups, which had similar ages. PSC patients had elevated gamma-glutamyl transferase (GGT) levels than patients with IBD (*P* = 0.005). Alkaline phosphatase (AP) and bilirubin levels were higher in PSC (*P* = 0.001 and *P* = 0.002, respectively) and PSC-IBD (*P* = 0.002 and *P* = 0.031, respectively) patients compared with IBD (see Table 1 for details).

**Table 1:** Details of the controls, patients with inflammatory bowel disease (IBD), patients with primary sclerosing cholangitis (PSC) and patients with PSC-IBD.

Characteristics	IBD	PSC	PSC-IBD	Controls
Number (females/males)	57 (26/31)	7 (2/5)	13 (6/7)	16 (9/7)
Age (years)	41.2 (19.1–69.9)	52.6 (25.7–69.7)	47.2 (18.2–65.8)	56.0 (29.3–78.1)
BMI (kg/m^2^)	24.1 (15.5–44.3)	n.d	24.1 (16.3–41.8)	n.d
C-reactive protein (mg/L)	2 (0–144)	n.d	2 (0–93)	n.d
Fecal calprotectin (µg/g)	58 (0–1616)	n.d	34 (0–222)	n.d
AST (U/L)	25 (10–35)	34 (15–177)	27 (16–161)	n.d
ALT (U/L)	18 (7–63)	27 (5–89)	25 (7–205)	n.d
GGT (U/L)	23 (8–100)	78 (14–345) ^%^	29 (10–458)	n.d
AP (U/L)	65 (38–142)	173 (70–537)	107 (35–587)	n.d
Bilirubin (mg/dL)	0.4 (0.1–1.9)	1.5 (0.4–14.0)	0.6 (0.2–21.3)	n.d
MELD Score	n.d	8 (6–14)	6 (6–12)	n.d

The median, minimum and maximum values are shown. Differences between the cohorts are described in the text.

Alanine aminotransferase (ALT), alkaline phosphatase (AP), aspartate aminotransferase (AST), body mass index (BMI), gamma-glutamyl transferase (GGT), model of end-stage liver disease (MELD), not determined (n.d.)).

### Ceramide and hexosylceramides in the serum of patients and controls

**Fig. 1:**
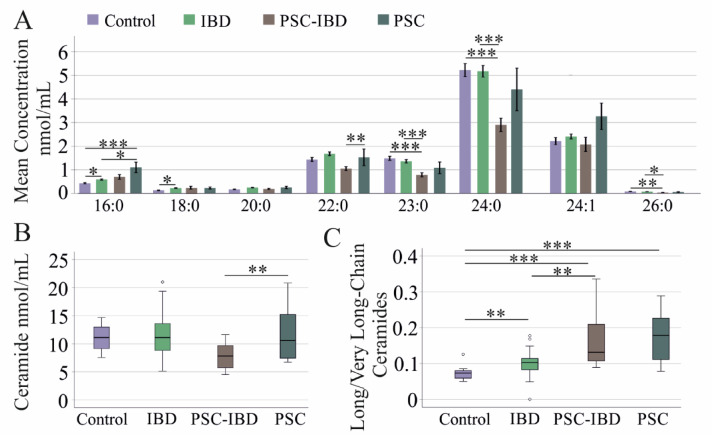
Serum ceramide species levels of controls, patients with inflammatory bowel disease (IBD), patients with primary sclerosing cholangitis and IBD (PSC-IBD) and patients with PSC. (**A**) Serum ceramide species levels. (**B**) Total ceramide levels in the serum of these patients. (**C**) Long-chain/very long-chain ceramide ratio of controls and patients with IBD, PSC-IBD and PSC. * *P* < 0.05, ** *P* < 0.01, *** *P* < 0.001.

Serum levels of eight ceramide (Cer) species and five hexosylceramide (HexCer) species were measured in patients with IBD, PSC-IBD, and PSC, as well as in healthy controls. The results showed that ceramide 18:1;O2/16:0 and 18:1;O2/18:0 levels were elevated in IBD patients compared to controls (Fig. 1A). No significant differences were observed in the remaining ceramide species between IBD patients and controls (Fig. 1A).

In contrast, PSC-IBD patients had reduced levels of ceramide 18:1;O2/23:0, 24:0, and 26:0 compared to both IBD patients and controls (Fig. 1A). Conversely, ceramide 18:1;O2/16:0 levels were elevated in PSC patients compared to both controls and IBD patients. Additionally, ceramide 18:1;O2/22:0 levels were higher in PSC patients than in PSC-IBD patients (Fig. 1A). PSC-IBD patients also exhibited significantly lower total ceramide levels compared to PSC patients (Fig. 1B).

Furthermore, the analysis revealed that the ratio of long-chain (LC) ceramides (C16–C20) to very long-chain (VLC) ceramides (C22–C26) was higher in IBD, PSC, and PSC-IBD patients compared to controls (Fig. 1C). Notably, this LC/VLC ratio was significantly higher in PSC-IBD patients than in IBD patients.

Total hexosylceramide levels did not differ significantly between the groups (*P* = 0.063). Similarly, total ceramide and hexosylceramide levels, as well as all measured ceramide species, showed no significant differences between the 40 CD patients and the 17 UC patients (*P* > 0.05 for all).

**Fig. 2:**
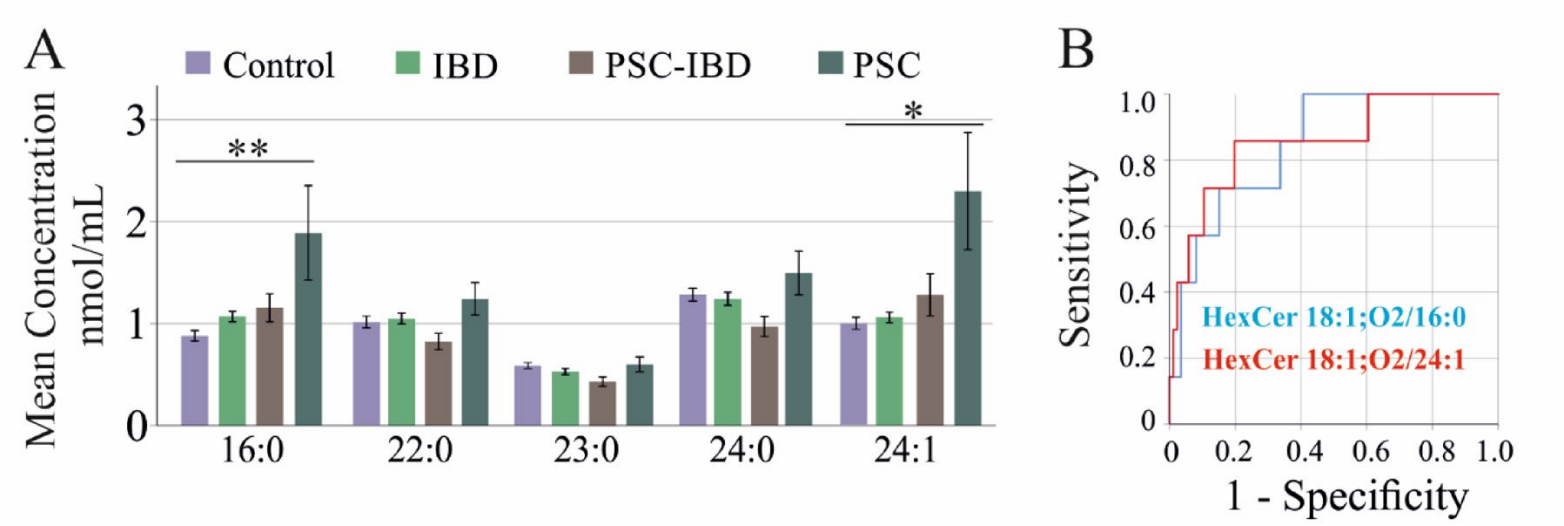
Serum hexosylceramide species levels of controls, patients with inflammatory bowel disease (IBD), patients with primary sclerosing cholangitis and IBD (PSC-IBD) and patients with PSC. (**A**) Hexosylceramide species levels of controls and patients with IBD, PSC-IBD and PSC. (**B**) Receiver operating characteristic curves for HexCer 18:1;O2/16:0 and HexCer 18:1;O2/24:1 for discriminating patients with PSC from all other groups. * *P* < 0.05, ** *P* < 0.01.

Hexosylceramide species HexCer 18:1;O2/16:0 and 24:1 were elevated in PSC patients compared to controls (Fig. 2A).

Receiver operating characteristic (ROC) curve analysis showed an area under the curve (AUROC) of 0.850 for HexCer 18:1;O2/16:0 (*P* = 0.028) and 0.857 for HexCer 18:1;O2/24:1 (*P* = 0.023), distinguishing PSC patients from all other groups (Fig. 2B).

### Effects of sex, age and BMI on ceramide and hexosylceramide levels in patients with IBD and controls

**Fig. 3:**
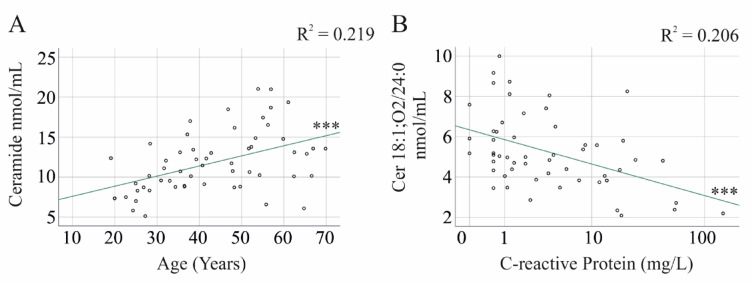
Correlation of ceramides with age and C-reactive protein in patients with inflammatory bowel disease (IBD). (**A**) Correlation of total ceramide levels with age. (**B**) Correlation of Cer 18:1;O2/24:0 with C-reactive protein. *** *P* < 0.001.

Lipid species did not differ between the nine female and seven male controls and showed no correlation with age. In IBD patients, ceramide 18:1;O2/22:0 (r = 0.493, *P* = 0.001), 23:0 (r = 0.397, *P* = 0.002), 24:0 (r = 0.569, *P* < 0.001), and total ceramide levels (r = 0.505, *P* < 0.001) were positively correlated with age (Fig. 3A). However, the LC/VLC ratio was not associated with age (*P* > 0.05).

Ceramide species levels in patients with IBD showed no significant correlation with BMI or sex. In the PSC-IBD cohort, neither lipid species correlated significantly with age or BMI. Male and female PSC-IBD patients had comparable ceramide and hexosylceramide levels. The PSC cohort was too small for meaningful statistical analysis.

### Correlation of ceramide and hexosylceramides with C-reactive protein and fecal calprotectin

Cer 18:1;O2/24:0 and HexCer 18:1;O2/22:0, 23:0, and 24:0 were negatively correlated with C-reactive protein (CRP) (Fig. 3B) and fecal calprotectin (Table 2). Additionally, ceramide 18:1;O2/26:0 showed a negative correlation with CRP (Table 2). All other ceramide species were not associated with CRP and fecal calprotectin levels. The negative correlation between the VLC ceramide species Cer 18:1;O2/24:0 with CRP and fecal calprotectin explains why the LC/VLC ceramide ratio was positively correlated with these markers of inflammation (Table 2). It should be noted that only one of the eight measured ceramide species and three of the five hexosylceramide species correlated with fecal calprotectin, suggesting that ceramide levels do mostly not change in active IBD.

**Table 2:** Spearman correlation of serum ceramide and hexosylceramide (HexCer) species and the long-chain to very long-chain ceramide ratio (LC/VLC) with C-reactive protein (CRP) and fecal calprotectin in patients with IBD and PSC-IBD.

Ceramide species	CRP	Calprotectin	CRP	Calprotectin
	IBD		PSC-IBD	
16:0	− 0.073	− 0.089	0.581	0.479
18:0	0.253	0.124	0.599	0.599
20:0	− 0.104	− 0.064	0.483	0.458
22:0	− 0.289	− 0.343	0.406	0.366
23:0	− 0.262	− 0.370	− 0.042	0.416
24:0	− 0.429*	− 0.429*	− 0.063	0.359
24:1	− 0.061	− 0.238	0.627	0.528
26:0	− 0.441*	− 0.319	− 0.193	0.218
HexCer 16:0	− 0.024	− 0.052	0.235	− 0.106
HexCer 22:0	− 0.496**	− 0.414*	− 0.459	− 0.493
HexCer 23:0	− 0.474**	− 0.391*	− 0.564	− 0.317
HexCer 24:0	− 0.532***	− 0.443**	− 0.630	− 0.451
HexCer 24:1	− 0.172	− 0.222	0.231	− 0.028
Total Ceramide	0.035	0.005	0.532	0.437
Total HexCer	0.006	0.014	0.053	− 0.225
LC/VLC	0.506**	0.512***	0.532	0.373

*P* < 0.05, ** *P* < 0.01, *** *P* < 0.001.

Total ceramide and hexosylceramide levels did not correlate with markers of inflammation (Table 2). In PSC-IBD, ceramide and hexosylcermide species, total ceramide and hexosylceramide levels, and the LC/VLC ceramide ratio were not associated with CRP or fecal calprotectin (Table 2). As previously mentioned, no statistical analyses were conducted for the small PSC subgroup.

### Relationships of ceramide and hexosylceramide species with stool consistency and the gastrointestinal symptom rating scale score

The Gastrointestinal Symptom Rating Scale (GSRS) was used to assess disease severity in IBD patients^36^. The study found that two IBD patients reported no symptoms, 36 had mild symptoms, 16 had moderate symptoms, and two experienced severe symptoms. Notably, the GSRS score of one patient was not recorded.

**Fig. 4:**
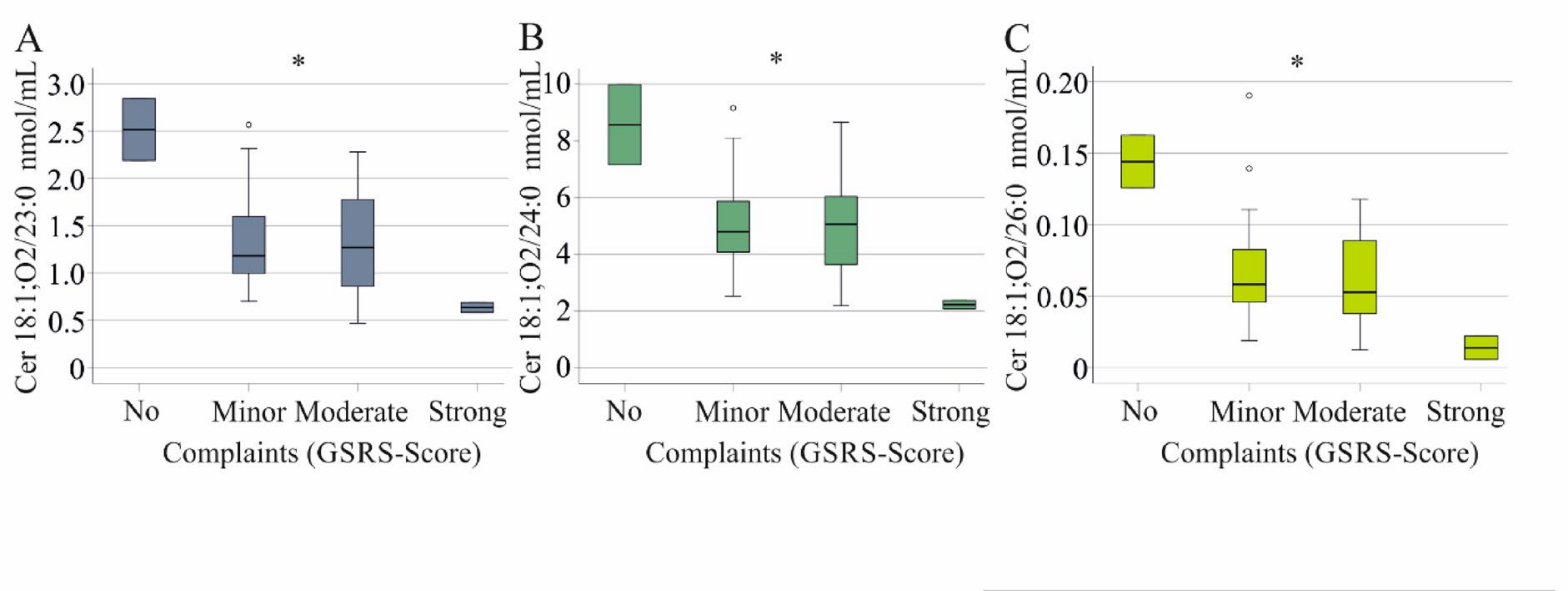
Ceramide species in relation to complaints of patients with inflammatory bowel disease. (**A**) Ceramide 18:1;O2/23:0, (**B**) Ceramide 18:1;O2/24:0 and (**C**) Ceramide 18:1;O2/26:0 in relation to complaints of patients with inflammatory bowel disease. * *P* < 0.05.

Analysis showed that ceramide 18:1;O2/23:0 (*p* = 0.028), ceramide 18:1;O2/24:0 (*p* = 0.031), and ceramide 18:1;O2/26:0 (*p* = 0.021) (Fig. 4A–C) were lower in patients with high GSRS scores than in those with low scores. The LC/VLC ratio did not change with increasing GSRS scores.

Ceramide 18:1;O2/23:0 and 18:1;O2/26:0 levels were not affected by rising fecal calprotectin levels (Table 2), suggesting that these associations are unrelated to intestinal inflammation. In contrast, ceramide 18:1;O2/24:0 negatively correlated with CRP and fecal calprotectin (Table 2), and its lower levels in patients with high GSRS scores may be linked to inflammation.

Ceramide and hexosylceramide species levels were similar across patients with constipation (n = 5), normal stool (n = 17), diarrhea (n = 27), and watery stool (n = 6) (*p* > 0.05 for all). Stool consistency was not recorded for two patients.

### Relationships between ceramides and hexosylceramides and disease localization and extraintestinal disease manifestations

In this patient cohort, the ileocaecal region was affected in eight patients with CD, while 29 had involvement of both the ileocaecal region and other parts of the gastrointestinal tract. In contrast, three patients had CD without ileocaecal involvement. Serum ceramide and hexosylceramide levels did not differ significantly between these groups (*P* > 0.05 for all).

Among patients with UC, one had isolated proctitis, three had proctosigmoiditis, two had left-sided colitis, and 10 had pancolitis. Disease localisation was not documented for one patient. No significant differences in serum ceramide or hexosylceramide levels were observed between these groups (*P* > 0.05 for all).

Among IBD patients, 34 had one or two extraintestinal manifestations. Arthralgia (22 patients), skin involvement (six patients), and ocular involvement (13 patients) were not associated with altered serum ceramide or hexosylceramide levels (*P* > 0.05 for all).

### Correlation of ceramide and hexosylceramides with measures of liver disease

In IBD patients, HexCer 18:1;O2/16:0 was inversely associated with bilirubin (Table 3).

**Table 3:** Spearman correlation of serum ceramide and hexosylceramide species with laboratory parameters of liver function in IBD patients and PSC-IBD patients.

Ceramide Species	AST	ALT	GGT	AP	Bilirubin	AST	ALT	GGT	AP	Bilirubin	MELD
	IBD					PSC-IBD					
16:0	0.052	0.001	0.050	0.174	0.024	0.642	0.569	0.785*	0.692	0.582	0.707
18:0	− 0.002	0.070	0.067	0.064	− 0.036	0.273	0.248	0.494	0.324	0.254	0.593
20:0	0.142	0.004	− 0.050	0.128	− 0.032	− 0.168	− 0.143	0.141	− 0.126	− 0.102	− 0.068
22:0	0.201	0.136	0.226	0.272	0.148	− 0.405	− 0.344	− 0.088	− 0.385	− 0.367	− 0.160
23:0	0.177	0.025	− 0.029	0.132	0.063	− 0.562	− 0.446	− 0.461	− 0.643	− 0.681	− 0.639
24:0	0.274	0.118	0.172	0.212	0.230	− 0.686	− 0.564	− 0.483	− 0.769*	− 0.803*	− 0.639
24:1	0.144	0.067	0.080	0.308	0.159	0.361	0.388	0.492	0.275	0.290	0.365
26:0	0.204	− 0.029	− 0.028	0.141	− 0.125	− 0.725	− 0.572	− 0.461	− 0.687	− 0.767*	− 0.593
HexCer 16:0	− 0.192	− 0.116	− 0.024	0.011	− 0.315*	0.736	0.638	0.878**	0.747*	0.717	0.730*
HexCer 22:0	0.153	0.051	0.008	− 0.057	0.055	− 0.039	− 0.151	0.135	0.176	0.077	− 0.137
HexCer 23:0	0.092	− 0.045	− 0.136	− 0.167	0.008	− 0.121	− 0.140	0.039	− 0.027	− 0.146	− 0.023
HexCer 24:0	0.277	0.112	− 0.015	− 0.018	0.113	− 0.143	− 0.220	− 0.088	0.016	− 0.113	− 0.023
HexCer 24:1	0.047	− 0.021	− 0.111	0.091	− 0.044	0.843**	0.666	0.796*	0.890**	0.808*	0.639
Total Ceramide	0.052	0.080	0.146	0.259	0.155	− 0.146	0.330	0.127	− 0.192	0.535	0.046
Total HexCer	0.072	− 0.001	− 0.024	0.004	− 0.036	0.482	− 0.074	0.666	0.659	− 0.204	0.365
LC/VLC	− 0.277	− 0.201	− 0.129	− 0.127	− 0.298	0.672	0.495	0.787*	0.874**	0.692	0.730

Alanine aminotransferase (ALT), alkaline phosphatase (AP), aspartate aminotransferase (AST), gamma-glutamyl transferase (GGT), hexosylceramide (HexCer), Model for End Stage Liver Disease (MELD). * *P* < 0.05, ** *P* < 0.01.

**Fig. 5:**
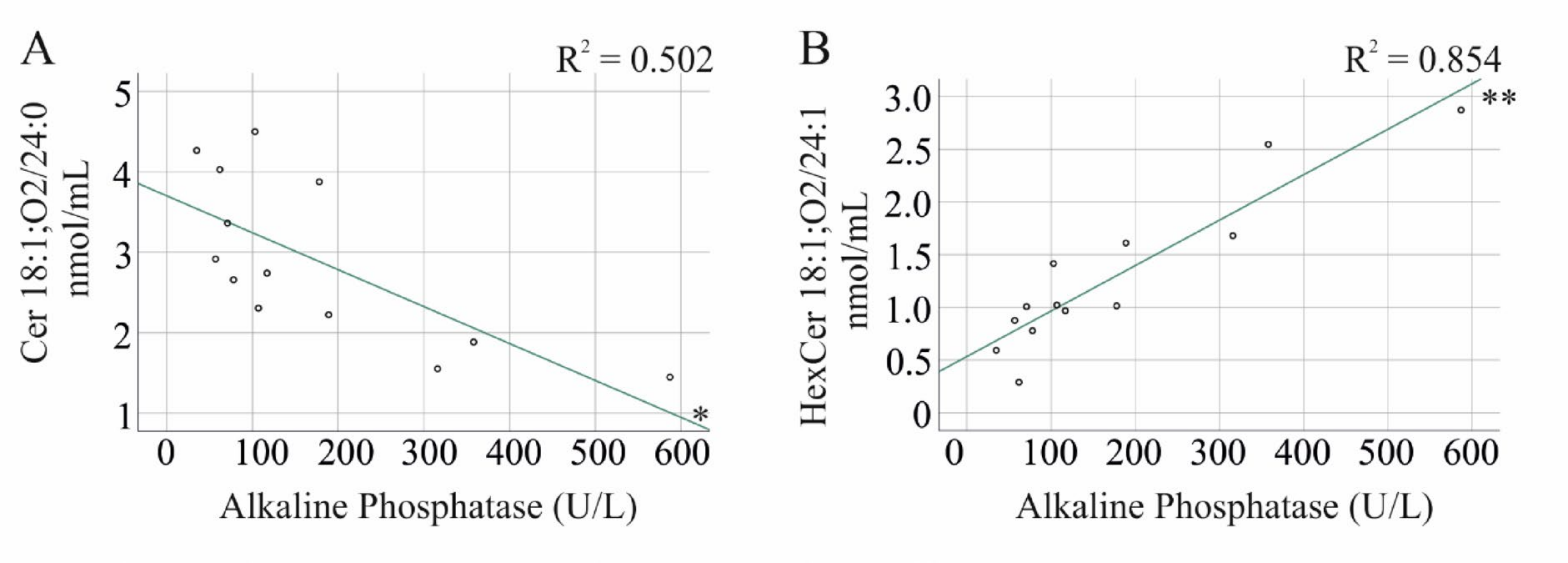
Correlation of Cer 18:1;O2/24:0 and HexCer 18:1;O2/24:1 with alkaline phosphatase levels in patients with primary sclerosing cholangitis and underlying inflammatory bowel disease. (**A**) Correlation of Cer 18:1;O2/24:0 with alkaline phosphatase activity. (**B**) Correlation of HexCer 18:1;O2/24:1 with alkaline phosphatase activity. * *P* < 0.05, ** *P* < 0.01.

In PSC-IBD patients, the following correlations were found:

1. Positive correlations with GGT: Cer 18:1;O2/16:0, HexCer 18:1;O2/16:0, HexCer 18:1;O2/24:1, LC/VLC ratio
2. Negative correlations with AP: Cer 18:1;O2/24:0
3. Positive correlations with AP: HexCer 18:1;O2/16:0 (Fig. 5A), HexCer 18:1;O2/24:1, LC/VLC ratio
4. Negative correlations with bilirubin: Cer 18:1;O2/24:0, Cer 18:1;O2/26:0
5. Positive correlations with bilirubin: HexCer 18:1;O2/24:1
6. Positive correlations with the MELD score: HexCer 18:1;O2/16:0
7. Positive correlations with AST: HexCer 18:1;O2/24:1

However, the PSC cohort was too small for meaningful statistical analysis.

### Comparison of studies having analysed systemic levels of ceramide and hexosylceramides in patients with IBD

Several studies have already measured serum/plasma ceramide species levels in controls and patients with IBD^37–41^ (Table 4).

**Table 4:** Comparison of serum ceramide and hexosylceramide species levels of patients with IBD and controls described in the current and in previous studies.

Study cohort	Current Study	Bazarganipour et al.^37^	Tefas et al.^41^	Ferru-Clement et al.^38^	Filimoniuk et al.^39^	Kubesch et al.^40^
Number of patients and controls (C)	57 IBD	98 UC	17 UC	200 CD	34 CD/ 39 UC	CD
	16 C	148 C	5 CD	200 C	24 C	
			24 C			
Age (years) of the patients	41	44	UC: 39	35	14	This was a congress abstract and further information was not given
			CD: 43			

Ceramide Species	Serum	Plasma	Serum	Serum	Serum	Serum
16:0	⇑	⇑		⇑		⇑
18:0	⇑	⇑	⇑		CD versus C ⇑	⇑
20:0		⇑			CD versus C ⇑	
					UC versus C ⇑	
22:0						
23:0				⇓		
24:0					UC versus C ⇑	⇑
24:1		⇑		⇑		
26:0						
HexCer 16:0		⇑				
HexCer 22:0						
HexCer 23:0				⇓		
HexCer 24:0						
HexCer 24:1		⇑				

⇑ higher in patients´ serum/plasma compared to controls, ⇓ lower in patients´ serum/plasma compared to controls.

These studies showed that LC ceramide species (C16–C20) were increased in the blood of patients with IBD (Table 4)^37–41^, and this was observed in patients with UC and CD^37–41^. The changes of VLC ceramide species (C22–C26) were less consistent and were found, increased and decreased in IBD^37–39^ (Table 4).

The association of ceramide species with fecal calprotectin as a biomarker for disease severity^42,43^ was less well studied. In patients with UC plasma Cer16:0, 18:0, 20:0 and 24:1 as well as GlcCer 16:0 and 24:1 of patients with mild and moderate/severe disease did not differ and were thus not strongly associated with disease severity (the lipid nomenclature was taken from the respective studies)^37^. In children with CD and UC, C18:0, 18:1 and 24:0-Cer did not correlate with fecal calprotectin and CRP levels. However, the negative correlation of C24:0-Cer with fecal calprotectin in patients with UC was almost significant (*P* = 0.07)^39^. Median CRP levels of CD patients were much higher compared to patients with UC, showing that increased inflammation alone does not trigger the decrease of C24:0-Cer. Moreover, C18:0-Cer was higher in CD and Cer18:1 in UC suggesting associations of these lipid species with disease etiology^39^. Higher fecal calprotectin levels in adult patients with IBD were associated with increased serum Cer(18:1;2O/22:0, 24:0 and 24:1) in 30 patients compared to the 45 patients with normal calprotectin levels. Serum CRP of these patients was nearly normal indicating less severe disease^44^. In our cohort, negative correlations of Cer 18:1;O2/24:0 and HexCer 18:1;O2/22:0, 23:0 and 24:0 with fecal calprotectin were observed.

These analyses show that the associations of individual ceramide species with fecal calprotectin need further studies. Large cohorts allowing for the separate analysis of disease entities and possible confounders, such as age, are needed. The LC/VLC ceramide ratio has not been calculated in these previous studies^37–41^, and may be a more robust measure compared to individual ceramide species.

## Discussion

Our study demonstrated that the serum LC/VLC ceramide ratio is elevated in IBD, PSC-IBD, and PSC patients compared to controls. (Fig. 1C). This ratio was positively correlated with inflammation in IBD and with cholestasis markers in PSC-IBD, indicating that an imbalance in LC and VLC ceramide levels is associated with disease severity.

In IBD patients, serum levels of Cer 18:1;O2/16:0 and 18:0 were elevated compared to controls, while VLC ceramide levels remained within the normal range (Fig. 1A). Notably, a higher level of LC ceramides in the blood of patients with IBD compared to healthy controls is in accordance with previous studies^37–41^. Whether VLC ceramides are also different between patients with IBD and controls is less clear, and increased and decreased levels have been described in IBD^37–39^.

VLC ceramides and hexosylceramide species were inversely correlated with CRP and fecal calprotectin in our study. In patients with UC, plasma LC and VLC ceramides of patients with mild and moderate/severe disease were similar and thus were not associated with disease severity^37^. This shows that whereas evidence for an increase of LC species is convincing^37–41^ the regulation of VLC species and the association of ceramide species with disease severity needs further study.

Serum ceramide and hexosylceramide levels did not differ between UC and CD patients in our study. This is in contrast to a previous analysis showing higher levels of Cer (18:1;2O/24:1) in patients with CD^44^. Moreover, higher C18:0-Cer levels in CD and C18:1-Cer in UC were also described^39^. This study included 34 cases with CD, 39 cases with UC and 24 controls and was not much larger than our cohort. Whether the small cohort sizes contributed to these inconsistent results^45^ needs further investigation. Studies in larger cohorts are needed to clarify whether ceramide species levels differ between patients with CD and UC.

Inflammation in UC is restricted to the colon, and can affect any part of the gastrointestinal tract in patients with CD^2,46^. Disease localisation in UC and CD was not linked to altered serum ceramide levels in the current analysis. However, the number of our patients for this analysis was small, and studies in larger cohorts, which can account for disease localization and disease severity, have to finally resolve this issue.

There is strong evidence that Cer 18:1;O2/16:0 levels increase during inflammation, while Cer 18:1;O2/24:0 levels decrease^47^. However, rather than an overall increase in ceramide levels, it is the imbalance between LC and VLC ceramides that is detrimental to cells^7,48^. In IBD, LC ceramide levels were elevated^37–41^, whereas in PSC-IBD, VLC ceramide levels were reduced, leading to a higher LC/VLC ratio in both. Additionally, VLC ceramide and VLC hexosylceramide species were negatively associated with markers of intestinal inflammation in IBD patients. In PSC-IBD patients, LC ceramides and LC hexosylceramides were positively correlated with laboratory markers of cholestatic liver disease. These findings align with previous studies highlighting the protective role of VLC ceramides and the potentially harmful effects of LC ceramide species^12,13,49^. However, whether an increased LC/VLC ceramide ratio is just a biomarker for disease severity or is actively involved in disease pathogenesis has not been clarified^3^.

Notably, VLC ceramides Cer 18:1;O2/23:0, 24:0, and 26:0 decreased as symptom severity increased in IBD patients (Fig. 4), with only Cer 18:1;O2/24:0 showing a negative correlation with fecal calprotectin (Table 2). These findings suggest that lower Cer 18:1;O2/23:0 and 26:0 levels are associated with symptom severity, independent of intestinal inflammation. Notably, ceramide and hexosylceramide species levels were not related to diarrhea, which is also an item of the GSRS.

Contrasting the finding in human IBD, a previous study demonstrated that an increase in saturated VLC ceramides drove the proinflammatory effects of IL-10 deficiency in murine macrophages^18^. Cer 22:0 and Cer 24:0 species further increased the expression of various cytokines and chemokines in macrophages activated by toll-like receptor 2 ligand^18^. In patients with PSC-IBD, serum Cer d18:1;O2/24:0 was markedly decreased in comparison to patients with IBD (Fig. 1A), whereas CRP and fecal calprotectin levels between these groups were similar (Table 1). This shows that the depletion of serum Cer d18:1;O2/24:0 is not associated with increased inflammation. Whether this is a compensatory mechanism and CRP and fecal calprotectin would be higher without the decline of Cer d18:1;O2/24:0 needs further study. It should be noted that the final concentration of the ceramides used to incubate macrophages was 30 µM^18^, which is higher than the ceramide levels measured in serum.

Notably, colonic inflammation in IL-10-deficient mice improved following the deletion of ceramide synthase 2, which is associated with lower VLC ceramide levels^18^. In contrast, ceramide synthase 2 loss has been shown to exacerbate disease severity in azoxymethane/DSS colitis^50^. Deleting ceramide synthase 2 significantly reduced VLC ceramides while simultaneously increasing LC ceramides in the colon and plasma of these mice^50^. However, ceramide synthase 2 knockout mice exhibit multiple severe defects, highlighting the need for tissue-specific knockout models to better understand its role^51,52^.

PSC is a rare disease closely linked to IBD^28,53^. To date, only one study has analysed the serum lipidome of PSC patients and controls, reporting normal serum ceramide levels in PSC patients^54^. However, this study did not distinguish between PSC with and without underlying IBD^54^. Our analysis revealed that PSC patients had higher levels of Cer 18:1;O2/22:0 and total ceramides compared to PSC-IBD patients (Fig. 1A). Additionally, Cer 18:1;O2/23:0, 24:0, and 26:0 were reduced in PSC-IBD patients compared to both controls and IBD patients, while no such differences were observed between PSC and IBD patients (Fig. 1A). It is still not clear whether IBD, PSC-IBD and PSC are different disease entities^27^ and the current analysis showed that at least the serum lipidome of these cohorts differs significantly.

Hexosylceramide levels remained within the normal range in IBD and PSC-IBD patients, PSC patients exhibited elevated serum levels of HexCer 18:1;O2/16:0 and 24:1 (Fig. 2A). These findings suggest that PSC-IBD is characterised by a reduction in VLC ceramides, whereas PSC patients maintain almost normal ceramide species levels, with an increase in Cer 18:1;O2/16:0, HexCer 18:1;O2/16:0 and 24:1 (Figs. 1A and 2A).

In PSC-IBD patients, correlation analysis revealed a consistent positive association between LC ceramides and hexosylceramides and markers of liver injury, while VLC ceramides predominantly showed negative associations. Notably, HexCer 18:1;O2/24:1 was an exception, demonstrating a strong positive correlation with cholestasis markers in PSC-IBD patients (Table 3). This lipid was significantly elevated in PSC patients, who also had higher levels of GGT, AP, and bilirubin compared to those with PSC-IBD, further supporting its association with cholestasis.

Importantly, ceramide and hexosylceramide species showed no correlation with liver disease markers in IBD patients (Table 3), suggesting that these associations are not driven by a direct effect of liver disease on serum lipid levels. Consistently, serum ceramide species did not correlate with liver disease markers in patients with hepatitis C virus infection, and significant reductions in nearly all ceramide species were only observed in cases of liver cirrhosis^55^. These findings highlight the potential role of HexCer 18:1;O2/24:1 as a biomarker for cholestasis and emphasize the need for further research to elucidate the underlying mechanisms driving these associations.

In IBD patients, saturated ceramide species and total serum ceramide levels showed a positive correlation with age (Fig. 3A). Similarly, C16:0, C18:0, and C24:1 ceramide levels were positively associated with age in a separate cohort^56^, highlighting the need for further research into the role of specific ceramide species in ageing. Notably, sex and BMI had no significant impact on ceramide and hexosylceramide levels, suggesting that these factors do not confound lipid analysis in IBD and PSC-IBD. However, a sex-specific analysis in a larger cohort of patients and controls is required to prove this assumption. Obesity is also a growing problem in IBD^57^ and may be related to an altered lipid profile in these patients, which has to be studied in the future.

This study has several limitations. The PSC and PSC-IBD subgroups were small, and larger cohorts are needed to validate these findings. The IBD cohort was too small to perform disease entity-specific analysis. Here, only a single serum sample per patient was analysed. Prospective studies collecting blood before and during therapy of patients with IBD, PSC and PSC-IBD will show whether the LC/VLC ceramide ratio is of diagnostic and prognostic relevance. Additionally, as a single-centre study conducted in southern Germany, further research is required to confirm its relevance to broader patient populations.

## Conclusions

Our study is the first to compare serum ceramide and hexosylceramide species in patients with IBD, PSC-IBD, and PSC. All three diseases are associated with an elevated LC/VLC ceramide ratio. Notably, the increase in serum hexosylceramide species is a distinctive feature of PSC and merits further investigation as both a pathophysiological marker and a potential non-invasive diagnostic tool.

## Data Availability

Original research data can be obtained from the corresponding author Christa Buechler upon request.

## Abbreviations

ALT: Alanine aminotransferase
AP: Alkaline phosphatase
AST: Aspartate aminotransferase
Cer: Ceramide
CD: Crohn’s disease
DSS: Dextran sulfate sodium
GGT: Gamma-glutamyl transferase
HexCer: Hexosylceramide
IBD: Inflammatory bowel disease
PSC: Primary sclerosing cholangitis
UC: Ulcerative colitis

## References

[1] Seyedian, S. S., Nokhostin, F. & Malamir, M. D. A review of the diagnosis, prevention, and treatment methods of inflammatory bowel disease. *J. Med. Life* **12**, 113–122. 10.25122/jml-2018-0075 (2019). 31406511 10.25122/jml-2018-0075 PMC6685307

[2] Gajendran, M., Loganathan, P., Catinella, A. P. & Hashash, J. G. A comprehensive review and update on Crohn’s disease. *Dis. Mon.* **64**, 20–57. 10.1016/j.disamonth.2017.07.001 (2018). 28826742 10.1016/j.disamonth.2017.07.001

[3] Doll, C. L. & Snider, A. J. The diverse roles of sphingolipids in inflammatory bowel disease. *FASEB J.* **38**, e23777. 10.1096/fj.202400830R (2024). 38934445 10.1096/fj.202400830R PMC467036

[4] Aldars-Garcia, L., Chaparro, M. & Gisbert, J. P. Systematic review: The gut microbiome and its potential clinical application in inflammatory bowel disease. *Microorganisms* **9**, 977. 10.3390/microorganisms9050977 (2021). 33946482 10.3390/microorganisms9050977 PMC8147118

[5] Park, W. J., Song, J. H., Kim, G. T. & Park, T. S. Ceramide and sphingosine 1-phosphate in liver diseases. *Mol. Cells* **43**, 419–430. 10.14348/molcells.2020.0054 (2020). 32392908 10.14348/molcells.2020.0054 PMC7264474

[6] Hajduch, E., Lachkar, F., Ferre, P. & Foufelle, F. Roles of ceramides in non-alcoholic fatty liver disease. *J. Clin. Med.* **10**, 792. 10.3390/jcm10040792 (2021). 33669443 10.3390/jcm10040792 PMC7920467

[7] Buechler, C. & Aslanidis, C. Role of lipids in pathophysiology, diagnosis and therapy of hepatocellular carcinoma. *BBA-Mol. Cell Biol. L.* **1865**, 158658. 10.1016/j.bbalip.2020.158658 (2020). 10.1016/j.bbalip.2020.158658 32058031

[8] Ponnusamy, S. et al. Sphingolipids and cancer: ceramide and sphingosine-1-phosphate in the regulation of cell death and drug resistance. *Fut. Oncol.* **6**, 1603–1624. 10.2217/fon.10.116 (2010). 10.2217/fon.10.116 PMC3071292 21062159

[9] Peters, L., Kuebler, W. M. & Simmons, S. Sphingolipids in atherosclerosis: Chimeras in structure and function. *Int. J. Mol. Sci.* **23**, 11948. 10.3390/ijms2319119485 (2022). 36233252 10.3390/ijms231911948 PMC9570378

[10] Montgomery, M. K. et al. Regulation of glucose homeostasis and insulin action by ceramide acyl-chain length: A beneficial role for very long-chain sphingolipid species. *Biochim. Biophys. Acta* **1828–1839**, 2016. 10.1016/j.bbalip.2016.08.016 (1861). 10.1016/j.bbalip.2016.08.016 27591968

[11] Park, J. W., Park, W. J. & Futerman, A. H. Ceramide synthases as potential targets for therapeutic intervention in human diseases. *Biochim. Biophys. Acta* **671–681**, 2014. 10.1016/j.bbalip.2013.08.019 (1841). 10.1016/j.bbalip.2013.08.019 24021978

[12] Ho, Q. W. C., Zheng, X. & Ali, Y. Ceramide acyl chain length and its relevance to intracellular lipid regulation. *Int. J. Mol. Sci.* **23**, 9697. 10.3390/ijms23179697 (2022). 36077094 10.3390/ijms23179697 PMC9456274

[13] Peterson, L. R. et al. Ceramide remodeling and risk of cardiovascular events and mortality. *J. Am. Heart Assoc.* **7**, e007931. 10.1161/JAHA.117.007931 (2018). 29728014 10.1161/JAHA.117.007931 PMC6015315

[14] Scheffel, M. J. et al. Adoptive transfer of ceramide synthase 6 deficient splenocytes reduces the development of colitis. *Sci. Rep.* **7**, 15552. 10.1038/s41598-017-15791-x (2017). 29138469 10.1038/s41598-017-15791-x PMC5686186

[15] Helke, K. et al. Ceramide synthase 6 deficiency enhances inflammation in the DSS model of colitis. *Sci. Rep.* **8**, 1627. 10.1038/s41598-018-20102-z (2018). 29374263 10.1038/s41598-018-20102-z PMC5786068

[16] El-Hindi, K. et al. T-cell-specific CerS4 depletion prolonged inflammation and enhanced tumor burden in the AOM/DSS-induced CAC model. *Int. J. Mol. Sci.* **23**, 1866. 10.3390/ijms23031866 (2022). 35163788 10.3390/ijms23031866 PMC8837088

[17] Abramczyk, J., Milkiewicz, M., Hula, B., Milkiewicz, P. & Kempinska-Podhorodecka, A. The role of hsa-miR-125b-5p interaction with S1P/ceramide axis in the potential development of inflammation-associated colon cancer in primary sclerosing cholangitis. *Int. J. Mol. Sci.* **24**, 9175. 10.3390/ijms24119175 (2023). 37298127 10.3390/ijms24119175 PMC10252877

[18] York, A. G. et al. IL-10 constrains sphingolipid metabolism to limit inflammation. *Nature* **627**, 628–635. 10.1038/s41586-024-07098-5 (2024). 38383790 10.1038/s41586-024-07098-5 PMC10954550

[19] Coant, N. & Hannun, Y. A. Neutral ceramidase: Advances in mechanisms, cell regulation, and roles in cancer. *Adv. Biol. Regul.* **71**, 141–146. 10.1016/j.jbior.2018.10.005 (2019). 30389354 10.1016/j.jbior.2018.10.005 PMC6347532

[20] Snider, A. J. et al. Loss of neutral ceramidase increases inflammation in a mouse model of inflammatory bowel disease. *Prostaglandins Other. Lipid. Mediat.* **99**, 124–130. 10.1016/j.prostaglandins.2012.08.003 (2012). 22940715 10.1016/j.prostaglandins.2012.08.003 PMC3661865

[21] Sandborn, W. J. et al. Etrasimod as induction and maintenance therapy for ulcerative colitis (ELEVATE): Two randomised, double-blind, placebo-controlled, phase 3 studies. *Lancet* **401**, 1159–1171. 10.1016/S0140-6736(23)00061-2 (2023). 36871574 10.1016/S0140-6736(23)00061-2

[22] Espaillat, M. P. et al. Loss of acid ceramidase in myeloid cells suppresses intestinal neutrophil recruitment. *FASEB J.* **32**, 2339–2353. 10.1096/fj.201700585R (2018). 29259036 10.1096/fj.201700585R PMC6207279

[23] Ishibashi, Y., Kohyama-Koganeya, A. & Hirabayashi, Y. New insights on glucosylated lipids: Metabolism and functions. *Biochim. Biophys. Acta* **1475–1485**, 2013. 10.1016/j.bbalip.2013.06.001 (1831). 10.1016/j.bbalip.2013.06.001 23770033

[24] Torretta, E. et al. Severity of COVID-19 patients predicted by serum sphingolipids signature. *Int. J. Mol. Sci.* **22**, 10198. 10.3390/ijms221910198 (2021). 34638539 10.3390/ijms221910198 PMC8508132

[25] Berkowitz, L., Salazar, C., Ryff, C. D., Coe, C. L. & Rigotti, A. Serum sphingolipid profiling as a novel biomarker for metabolic syndrome characterization. *Front. Cardiovasc. Med.* **9**, 1092331. 10.3389/fcvm.2022.1092331 (2022). 36578837 10.3389/fcvm.2022.1092331 PMC9791223

[26] Vesterhus, M. & Karlsen, T. H. Emerging therapies in primary sclerosing cholangitis: Pathophysiological basis and clinical opportunities. *J. Gastroenterol.* **55**, 588–614. 10.1007/s00535-020-01681-z (2020). 32222826 10.1007/s00535-020-01681-z PMC7242240

[27] van Munster, K. N., Bergquist, A. & Ponsioen, C. Y. Inflammatory bowel disease and primary sclerosing cholangitis: One disease or two?. *J. Hepatol.*10.1016/j.jhep.2023.09.031 (2023). 37940453 10.1016/j.jhep.2023.09.031

[28] Karlsen, T. H., Folseraas, T., Thorburn, D. & Vesterhus, M. Primary sclerosing cholangitis - A comprehensive review. *J. Hepatol.* **67**, 1298–1323. 10.1016/j.jhep.2017.07.022 (2017). 28802875 10.1016/j.jhep.2017.07.022

[29] Bedke, T. et al. Protective function of sclerosing cholangitis on IBD. *Gut* **73**, 1292–1301. 10.1136/gutjnl-2023-330856 (2024). 38839272 10.1136/gutjnl-2023-330856 PMC11287650

[30] Sun, X. et al. Untargeted lipidomics analysis in women with intrahepatic cholestasis of pregnancy: A cross-sectional study. *Bjog-an Int. J. Obstetrics Gynaecol.*10.1111/1471-0528.17026 (2021). 10.1111/1471-0528.17026 34797934

[31] Kucharzik, T., Dignass, A. & Siegmund, B. Aktualisierung der S3-Leitlinie Colitis ulcerosa 2019. *Z. Gastroenterol.* **57**, 1279–1280. 10.1055/a-1015-7048 (2019). 31739372 10.1055/a-1015-7048

[32] Sturm, A. et al. ECCO-ESGAR Guideline for Diagnostic Assessment in IBD Part 2: IBD scores and general principles and technical aspects. *J. Crohns Colitis* **13**, 273–284. 10.1093/ecco-jcc/jjy114 (2019). 30137278 10.1093/ecco-jcc/jjy114

[33] Bligh, E. G. & Dyer, W. J. A rapid method of total lipid extraction and purification. *Can. J. Biochem. Physiol.* **37**, 911–917. 10.1139/o59-099 (1959). 13671378 10.1139/o59-099

[34] Liebisch, G. et al. Quantitative measurement of different ceramide species from crude cellular extracts by electrospray ionization tandem mass spectrometry (ESI-MS/MS). *J. Lipid Res.* **40**, 1539–1546 (1999). 10428992

[35] Liebisch, G., Lieser, B., Rathenberg, J., Drobnik, W. & Schmitz, G. High-throughput quantification of phosphatidylcholine and sphingomyelin by electrospray ionization tandem mass spectrometry coupled with isotope correction algorithm. *Biochim. Biophys. Acta* **1686**, 108–117. 10.1016/j.bbalip.2004.09.003 (2004). 15522827 10.1016/j.bbalip.2004.09.003

[36] Schafer, S. K. et al. Design and validation of a German version of the GSRS-IBS - An analysis of its psychometric quality and factorial structure. *BMC Gastroenterol.* **17**, 139. 10.1186/s12876-017-0684-8 (2017). 29202711 10.1186/s12876-017-0684-8 PMC5715554

[37] Bazarganipour, S. et al. The lipid status in patients with ulcerative colitis: Sphingolipids are disease-dependent regulated. *J. Clin. Med.* **8**, 971. 10.3390/jcm8070971 (2019). 31277430 10.3390/jcm8070971 PMC6678307

[38] Ferru-Clement, R. et al. Serum lipidomic screen identifies key metabolites, pathways, and disease classifiers in crohn’s disease. *Inflamm. Bowel Dis.* **29**, 1024–1037. 10.1093/ibd/izac281 (2023). 36662167 10.1093/ibd/izac281 PMC10320374

[39] Filimoniuk, A., Blachnio-Zabielska, A., Imierska, M., Lebensztejn, D. M. & Daniluk, U. Sphingolipid analysis indicate lactosylceramide as a potential biomarker of inflammatory bowel disease in children. *Biomolecules* **10**, 1083. 10.3390/biom10071083 (2020). 32708181 10.3390/biom10071083 PMC7408557

[40] Kubesch, A. et al. Investigation of the serum sphingolipid and ceramide profile in patients with Crohn’s disease at different stages of the disease. *J. Crohns Colitis* **19**, i359 (2025).

[41] Tefas, C., Ciobanu, L., Tantau, M., Moraru, C. & Socaciu, C. The potential of metabolic and lipid profiling in inflammatory bowel diseases: A pilot study. *Bosn J. Basic Med. Sci.* **20**, 262–270. 10.17305/bjbms.2019.4235 (2020). 31368421 10.17305/bjbms.2019.4235 PMC7202185

[42] Jukic, A., Bakiri, L., Wagner, E. F., Tilg, H. & Adolph, T. E. Calprotectin: From biomarker to biological function. *Gut* **70**, 1978–1988. 10.1136/gutjnl-2021-324855 (2021). 34145045 10.1136/gutjnl-2021-324855 PMC8458070

[43] van Rheenen, P. F., Van de Vijver, E. & Fidler, V. Faecal calprotectin for screening of patients with suspected inflammatory bowel disease: Diagnostic meta-analysis. *BMJ* **341**, c3369. 10.1136/bmj.c3369 (2010). 20634346 10.1136/bmj.c3369 PMC2904879

[44] Tien, N. T. N. et al. An exploratory multi-omics study reveals distinct molecular signatures of ulcerative colitis and Crohn’s disease and their correlation with disease activity. *J. Pharm. Biomed. Anal.* **255**, 116652. 10.1016/j.jpba.2024.116652 (2025). 39740478 10.1016/j.jpba.2024.116652

[45] Cao, Y., Chen, R. C. & Katz, A. J. Why is a small sample size not enough?. *Oncologist* **29**, 761–763. 10.1093/oncolo/oyae162 (2024). 38934301 10.1093/oncolo/oyae162 PMC11379640

[46] Gajendran, M. et al. A comprehensive review and update on ulcerative colitis(). *Dis. Mon.* **65**, 100851. 10.1016/j.disamonth.2019.02.004 (2019). 30837080 10.1016/j.disamonth.2019.02.004

[47] Garic, D., De Sanctis, J. B., Shah, J., Dumut, D. C. & Radzioch, D. Biochemistry of very-long-chain and long-chain ceramides in cystic fibrosis and other diseases: The importance of side chain. *Prog. Lipid Res.* **74**, 130–144. 10.1016/j.plipres.2019.03.001 (2019). 30876862 10.1016/j.plipres.2019.03.001

[48] Hartmann, D. et al. The equilibrium between long and very long chain ceramides is important for the fate of the cell and can be influenced by co-expression of CerS. *Int. J. Biochem. Cell Biol.* **45**, 1195–1203. 10.1016/j.biocel.2013.03.012 (2013). 23538298 10.1016/j.biocel.2013.03.012

[49] McNally, B. D. et al. Long-chain ceramides are cell non-autonomous signals linking lipotoxicity to endoplasmic reticulum stress in skeletal muscle. *Nat. Commun.* **13**, 1748. 10.1038/s41467-022-29363-9 (2022). 35365625 10.1038/s41467-022-29363-9 PMC8975934

[50] Oertel, S. et al. Ceramide synthase 2 deficiency aggravates AOM-DSS-induced colitis in mice: role of colon barrier integrity. *Cell Mol. Life Sci.* **74**, 3039–3055. 10.1007/s00018-017-2518-9 (2017). 28405720 10.1007/s00018-017-2518-9 PMC11107765

[51] Bickert, A. et al. Inactivation of ceramide synthase 2 catalytic activity in mice affects transcription of genes involved in lipid metabolism and cell division. *Biochim. Biophys. Acta Mol. Cell Biol. Lipids* **734–749**, 2018. 10.1016/j.bbalip.2018.04.006 (1863). 10.1016/j.bbalip.2018.04.006 29653252

[52] Imgrund, S. et al. Adult ceramide synthase 2 (CERS2)-deficient mice exhibit myelin sheath defects, cerebellar degeneration, and hepatocarcinomas. *J. Biol. Chem.* **284**, 33549–33560. 10.1074/jbc.M109.031971 (2009). 19801672 10.1074/jbc.M109.031971 PMC2785198

[53] Rabiee, A. & Silveira, M. G. Primary sclerosing cholangitis. *Transl. Gastroenterol. Hepatol.* **6**, 29. 10.21037/tgh-20-266 (2021). 33824933 10.21037/tgh-20-266 PMC7829069

[54] Banales, J. M. et al. Serum metabolites as diagnostic biomarkers for cholangiocarcinoma, hepatocellular carcinoma, and primary sclerosing cholangitis. *Hepatology* **70**, 547–562. 10.1002/hep.30319 (2019). 30325540 10.1002/hep.30319 PMC6767196

[55] Höring, M. et al. Serum ceramide species are associated with liver cirrhosis and viral genotype in patients with hepatitis C infection. *Int. J. Mol. Sci.* **23**, 9806. 10.3390/ijms23179806 (2022). 36077197 10.3390/ijms23179806 PMC9456360

[56] Kim, B. J. et al. Elevated ceramides 18:0 and 24:1 with aging are associated with hip fracture risk through increased bone resorption. *Aging (Albany NY)* **11**, 9388–9404. 10.18632/aging.102389 (2019). 31675352 10.18632/aging.102389 PMC6874435

[57] Singh, S., Dulai, P. S., Zarrinpar, A., Ramamoorthy, S. & Sandborn, W. J. Obesity in IBD: Epidemiology, pathogenesis, disease course and treatment outcomes. *Nat. Rev. Gastroenterol. Hepatol.* **14**, 110–121. 10.1038/nrgastro.2016.181 (2017). 27899815 10.1038/nrgastro.2016.181 PMC5550405

